# Low-Level Mercury Can Enhance Procoagulant Activity of Erythrocytes: A New Contributing Factor for Mercury-Related Thrombotic Disease

**DOI:** 10.1289/ehp.0901473

**Published:** 2010-03-23

**Authors:** Kyung-Min Lim, Sujin Kim, Ji-Yoon Noh, Keunyoung Kim, Won-Hee Jang, Ok-Nam Bae, Seung-Min Chung, Jin-Ho Chung

**Affiliations:** College of Pharmacy, Seoul National University, Seoul, Korea

**Keywords:** erythrocyte, mercury, phosphatidylserine exposure, procoagulant activity

## Abstract

**Background:**

Associations between cardiovascular diseases and mercury have been frequently described, but underlying mechanisms are poorly understood.

**Objectives:**

We investigate the procoagulant activation of erythrocytes, an important contributor to thrombosis, by low-level mercury to explore the roles of erythrocytes in mercury-related cardiovascular diseases.

**Methods:**

We used freshly isolated human erythrocytes and *ex vivo* and *in vivo* thrombosis models in rats to investigate mercury-induced procoagulant activity.

**Results:**

Prolonged exposure to low-dose mercuric ion (Hg^2+^; 0.25–5 μM for 1–48 hr) induced erythrocyte shape changes from discocytes to echinocytes to spherocytes, accompanied by microvesicle (MV) generation. These MVs and remnant erythrocytes expressed phosphatidylserine (PS), an important mediator of procoagulant activation. Hg^2+^ inhibited flippase, an enzyme that recovers PS into the inner leaflet of the cell membrane, and activated scramblase, an enzyme that alters lipid asymmetry in the cell membrane. Consistent with these activity changes, Hg^2+^ increased intracellular calcium and depleted ATP and protein thiol. A thiol supplement reversed Hg^2+^-induced MV generation and PS exposure and inhibited the increase in calcium ion (Ca^2+^) and depletion of ATP, indicating that free-thiol depletion was critical to Hg^2+^-mediated procoagulant activity. The procoagulant activity of Hg^2+^-treated erythrocytes was demonstrated by increased thrombin generation and endothelial cell adhesion. We further confirmed Hg^2+^-mediated procoagulant activation of erythrocytes in *ex vivo* and *in vivo* rat thrombosis models, where Hg^2+^ treatment (0.5–2.5 mg/kg) increased PS exposure and thrombus formation significantly.

**Conclusion:**

This study demonstrated that mercury could provoke procoagulant activity in erythrocytes through protein-thiol depletion–mediated PS exposure and MV generation, ultimately leading to enhanced thrombosis.

Mercury (Hg) is a heavy metal element widely distributed in the earth’s crust, seawater, freshwater, and air (Environment Canada 2004). Concomitantly with modern industrialization, human exposure to mercury has increased through anthropogenic mercury emissions from fuel combustion, municipal incinerators, and chemical industries. Mercury is considered a major environmental toxicant throughout the world (Agency for Toxic Substances and Disease Registry 2007; Mahaffey and Mergler 1998; Salonen et al. 2000; Valera et al. 2008), and much effort is being directed toward reducing environmental mercury pollution (Trasande et al. 2005). In a study of New York City adults, the mean blood mercury level was 2.73 μg/L, higher than blood lead or cadmium levels (McKelvey et al. 2007). Moreover, in that study, consumers of high-fish diets exhibited 3.7 times the blood mercury levels observed in those who reported consuming no fish. In another study, Akagi et al. (1995) reported that some people living in areas active in gold mining showed an extremely high level of blood mercury, close to 150 μg/L (~ 0.75 μM), raising a strong concern over the mercury exposure problem.

Mercury is harmless in insoluble form, but vapor or soluble forms such as inorganic mercury or methylmercury can be extremely toxic to humans. Most human mercury exposure occurs through inhalation of elemental mercury vapor released from dental amalgam and through the consumption of fish contaminated with methylmercury (Berlin et al. 2007). Once inhaled, elemental mercury vapor is rapidly accumulated into erythrocytes and undergoes oxidation to the mercuric ion (Hg^2+^) by catalase. Orally absorbed methylmercury is preferentially distributed into erythrocytes (~ 90%) and slowly turns into Hg^2+^ through demethylation in the spleen and liver. It has been demonstrated that 6–15% of total mercury in the blood of populations consuming high-fish diets exists in the form of inorganic mercury (Berglund et al. 2005; Rodrigues et al. 2010). Because elemental mercury or methylmercury ultimately turns into Hg^2+^ in the body (Zalups 2000), mercuric salt has been commonly used to investigate the toxicity of mercury.

Mercury toxicity manifests mainly as neuronal disorders, immunotoxicity, and kidney damage (Clarkson et al. 2003); however, cardiovascular diseases (CVDs), including atherosclerosis, coronary heart diseases, pulmonary embolism, hypertension, and vessel obstruction, have been frequently described in relation to mercury exposure (Houston 2007; Virtanen et al. 2005, 2007). Previously, CVDs associated with mercury exposure have been assumed to occur as a consequence of renal effects of mercury, but increasing attention is also being given to direct effects of mercury on cardiovascular (CV) tissues, including blood vessels, endothelial cells (Wiggers et al. 2008), platelets (Macfarlane 1981), and erythrocytes (Suwalsky et al. 2000). However, the role of direct toxic effects of mercury on CV tissues in the pathogenesis of mercury-associated CVDs has not been clarified.

Well-characterized hemolytic and anemia-inducing effects of mercury suggest that the erythrocyte might be an important target of mercury (Zolla et al. 1997). Eisele et al. (2006) reported that low-level Hg^2+^ exposure can induce phosphatidylserine (PS) translocation to the external surface of the erythrocyte cell membrane (i.e., PS exposure) through the modulation of a clotrimazole-sensitive potassium ion (K^+^) channel. These authors described the PS-exposing effects of Hg^2+^ in relation to the apoptosis of erythrocytes. However, the implications of PS-externalized erythrocytes in procoagulant activation and subsequent CVDs were not addressed. In another study, alterations of the erythrocyte membrane, including PS exposure and PS-bearing microvesicle (MV) formation, were reported to be able to render erythrocytes procoagulant, enabling the active participation of erythrocytes in thrombosis (Chung et al. 2007). PS exposed on the surface of erythrocytes provides a site for the assembly of the prothrombinase and tenase complex, leading to efficient thrombin generation and ultimately to clotting (Zwaal et al. 1977; Zwaal and Schroit 1997). Furthermore, increased adhesion of PS-expressing erythrocytes to endothelial cells contributes to vasoocclusion (Closse et al. 1999). MVs generated from deformed erythrocytes through vesiculation also contribute to an acceleration of the coagulation cascade via strong procoagulant activity and by serving as a rich source of PS (Martinez et al. 2005).

In the present study, we discovered that both MV generation and PS exposure could be induced in human erythrocytes by low-dose Hg^2+^ (HgCl_2_, 0.25–5 μM). Of note, Hg^2+^-mediated PS exposure and MV generation could enhance thrombin generation and adhesion to vascular endothelial cells, which is considered a direct marker for procoagulant activity. We examined the mechanism underlying, and *in vivo* relevancy of, Hg^2+^-induced procoagulant activation of erythrocytes in an effort to gain insight into the CVDs associated with mercury exposure.

## Materials and Methods

### Materials

We purchased mercury chloride (HgCl_2_), calcium chloride (CaCl_2_), EDTA, bovine serum albumin (BSA), KH_2_PO_4_, NaCl, Na_2_HPO_4_, KCl, Tris/HCl, MgCl_2_, NaH_2_PO_4_, dextrose, sodium citrate, Tris-base, NaHCO_3_, dimethyl sulfoxide, ethanol, Triton X-100, trichloroacetic acid, Tris-acetate, ATP bioluminescent assay kit, iodoacetic acid, adenosine, ethylene glycol tetraacetic acid (EGTA), acetic acid, purified human thrombin, and calcium ionophore A23187 from Sigma Chemical Co. (St. Louis, MO, USA). Other chemicals were of the highest grade available.

### Preparation of human erythrocytes

This study was approved by the Institutional Review Board at the Seoul National University/Health Service Center, and all subjects provided written informed consent. On the day of each experiment, human blood was obtained from healthy male donors (18–25 years of age) into Vacutainers containing acid citrate dextrose (Becton Dickinson, San Diego, CA, USA). Platelet-rich plasma and buffy coat were removed by aspiration after centrifugation at 200 × *g* for 15 min. Packed erythrocytes were washed three times with phosphate-buffered saline (PBS; 1 mM KH_2_PO_4_, 154 mM NaCl, 3 mM Na_2_HPO_4_, pH 7.4) and once with Tris buffer (15 mM Tris-HCl, 150 mM NaCl, 5 mM KCl, 2 mM MgCl_2_, pH 7.4) or Ringer solution (125 mM NaCl, 5 mM KCl, 1 mM MgSO_4_, 32 mM HEPES, 5 mM glucose, pH 7.4). Washed erythrocytes were resuspended in Tris buffer or Ringer solution to a cell concentration of 5 × 10^7^ cells/mL, and the final CaCl_2_ concentration was adjusted to 1 mM before use.

### Microscopic observation using scanning electron microscopy and confocal microscopy

After fixation with 2% glutaraldehyde solution for 1 hr at 4°C, the erythrocytes were centrifuged and washed three times with PBS, followed by postfixation with 1% osmium tetroxide for 30 min at room temperature. After washing with PBS several times, the samples were dehydrated serially with 50%, 75%, 90%, and 100% ethanol. After drying and coating with gold, the images were observed on a scanning electron microscope (SEM; JEOL, Tokyo, Japan). For confocal microscopy, 200 μL erythrocyte suspension was added and attached for 1 hr to an eight-chambered coverslip (Lab-Tek; Nalge Nunc Inc., Naperville, IL) that had been coated with 0.1 mg/mL poly-l-lysine. After washing the coverslip three times with Tris-buffered saline (TBS) containing 2% BSA, erythrocytes were stained with TBS buffer containing anti–glycophorin-A–FITC for 30 min and washed three times again. Erythrocytes were then incubated with vehicle (TBS) or Hg^2+^ and observed using confocal microscopy equipped with an argon laser (Leica, Wetzlar, Germany). Excitation and emission filters were set at 488 nm and 550–600 nm, respectively.

### Flow cytometric analysis of PS exposure and cytosolic calcium in erythrocytes

We used fluorescein isothiocyanate (FITC)-labeled annexin V (annexin V-FITC; Pharmingen, San Diego, CA, USA) as a marker for PS positivity and phycoerythrin-labeled monoclonal antibody against human glycophorin A (anti-glycophorin A–RPE; Dako Cytomation, Glostrub, Denmark) to identify erythrocytes. Negative controls for annexin V binding were stained with annexin V-FITC in the presence of 2.5 mM EDTA instead of 2.5 mM CaCl_2_. For detection of intracellular calcium increase, erythrocytes were loaded with 3 μM Fluo-4-AM (Molecular Probes, Eugene, OR, USA) for 1 hr at 37°C in the dark. Subsequently, the cells were washed twice and then resuspended in Tris buffer to a final concentration of 5 × 10^7^ cells/mL with 1 mM of CaCl_2_. For the confirmation of interference of Fluo-4 calcium signal by Hg^2+^, *N*,*N*,*N′*,*N*-tetrakis(2-pyridylmethyl)ethylenediamine (TPEN; 100 μM; Sigma Chemical Co.) was added for 5 min to extract Fluo-4–bound Hg^2+^. To quench extracellular calcium, 3 mM EGTA was added to the erythrocyte suspension. Samples were analyzed on a FACScalibur flow cytometer (Becton Dickinson). Data from 10,000 events were collected and analyzed using CellQuest Pro software (Becton Dickinson).

### Phospholipid translocation measurement

We measured phospholipid translocation according to the method described by Hilarius et al. (2004). Briefly, erythrocytes (5 × 10^7^ cells/mL) were incubated with Hg^2+^ and then loaded with 0.5 μM 1-Palmitoyl-2-[6-[(7-nitro-2-1,3-benzoxadiazol-4-yl)amino]hexanoyl]-sn-glycero-3-phospho-l-serine (C_6_-NBD-PS; Avanti Polar Lipids, Alabaster, AL, USA) for the flippase activity assay or 1-oleoyl-2-[6-[(7-nitro-2–1,3-benzoxadiazol-4-yl)amino]hexanoyl]-sn-glycero-3-phosphocholine (C_6_-NBD-PC; Avanti Polar Lipids) for the scramblase activity assay. Aliquots of cell suspension were removed at the indicated time intervals, placed in cold Tris buffer, and incubated on ice for 10 min in the presence or absence of 1% BSA. The amount of internalized probe was determined by comparing the fluorescence intensity associated with the cells before and after back-extraction by BSA. Samples were analyzed on the FACScalibur flow cytometer.

### Intracellular ATP level measurement

After incubation with Hg^2+^, erythrocytes were washed and resuspended in Tris buffer containing 1 mM CaCl_2_. The aliquot was mixed vigorously with 10% trichloroacetic acid solution and TAE buffer (100 mM Tris-acetate, 2 mM EDTA, pH 7.8) and then cooled on ice for 20 min. The sample was centrifuged, and the aliquot of resultant supernatant was mixed with cold TAE buffer. Samples were adapted to the luciferin/luciferase assay in a Luminoskan microplate reader (Labsystems, Franklin, MA, USA) using an ATP assay kit (Sigma). We calculated the ATP concentrations based on the ATP standard curve.

### Protein thiol level measurement

We determined protein thiol concentrations using a modified assay based on the colorimetric method described by Di Monte et al. (1984). After incubation with various concentrations of Hg^2+^, erythrocytes were centrifuged at 7,000 × *g* for 1 min, and the supernatant was removed. The pellet was resuspended with lysis buffer (5 mM sodium phosphate, pH 8) and incubated on ice for 30 min. Total lysate was resuspended with 5% perchloric acid 2:5 and then centrifuged at 7,000 × *g* for 2 min. The pellet was solubilized in 1 mL Tris-EDTA buffer (0.5 mM Tris-HCl, 5 mM EDTA, pH 7.6) containing 1% sodium dodecyl sulfate. 5,5′-Dithio-bis-(2-nitrobenzoic acid) (DTNB; 250 μM) was added to the samples, and the change of absorbance was measured at 412 nm. The content of protein thiol was calculated on the basis of a glutathione calibration curve and divided by the protein content, which was measured by Bio-Rad protein assay kit (Bio-Rad, Hercules, CA, USA).

### Prothrombinase assay

After incubation with Hg^2+^ for 4 hr, erythrocytes were incubated with 5 nM factor Xa and 10 nM factor Va (both from Hematologic Technologies Inc., Essex Junction, VT, USA) in Tyrode buffer (134 mM NaCl, 10 mM HEPES, 5 mM glucose, 2.9 mM KCl, 1 mM MgCl_2_, 12 mM NaHCO_3_, 0.34 mM Na_2_HPO_4_, 0.3% BSA, 2 mM CaCl_2_, pH 7.4) for 3 min at 37°C. Thrombin formation was initiated by adding 2 μM prothrombin. Exactly 3 min after addition of prothrombin, an aliquot of the suspension was transferred to a tube containing stop buffer (50 mM Tris-HCl, 120 mM NaCl, 2 mM EDTA, pH 7.9). Thrombin activity was determined using the chromogenic substrate S2238 (chromogenic substrate for thrombin; Chromogenix, Milano, Italy). We calculated the rate of thrombin formation from the change in absorbance at 405 nm using a calibration curve generated with active-site–titrated thrombin.

### Measurement of thrombin generation in plasma

We measured thrombin generation in plasma according to the method described by Peyrou et al. (1999). Briefly, Tris buffer- or Hg^2+^-treated erythrocytes were added to plasma, and under gentle magnetic stirring, thrombin formation was initiated by adding human recombinant tissue factor (Recombiplastin; Instrumentation Laboratory, Lexington, MA, USA) diluted (1:3,200) in Tris buffer containing 100 mM CaCl_2_ to the mixture. After 10 min, the aliquots were collected and transferred to Tris buffer containing 20 mM EDTA. The thrombin concentration was obtained as described above for the prothrombinase assay.

### Adherence of erythrocytes to human umbilical vein endothelial cells (HUVEC)

The HUVEC (three passages) were maintained in EGM (endothelial cell growth media)kit (Clonetics, Walkersville, MD) at 37°C in a 95% air/5% CO_2_ incubator. Before the experiments, 1 × 10^5^ cells were seeded into a T25 flask and grown for 5 days. Erythrocyte adherence to HUVEC was measured using a modification of the method described by Chung et al. (2007). PBS- or Hg^2+^-treated erythrocytes were washed twice and resuspended in endothelial basal medium (EBM)-2 (Clonetics) to a cell concentration of 5 × 10^7^ cells/mL. After HUVEC were washed twice with EBM-2 to remove media, the erythrocytes were layered onto a confluent HUVEC monolayer and incubated for 45 min at 37°C. After the incubation, the flask was rinsed three times with EBM-2 to remove nonadherent erythrocytes. The number of adherent erythrocytes was counted on a light microscope. The experiments were performed in triplicate, and 28 fields were selected randomly for counting erythrocytes.

### *Ex vivo* PS exposure measurement and venous thrombosis animal model

We used male Sprague-Dawley rats (SamTako Co., Osan, Korea) weighing 180–250 g for animal studies. For the measurement of PS exposure, animals were injected intravenously with saline (vehicle) or HgCl_2_ (bolus, 1.0 and 2.5 mg/kg/0.3 mL) into a left femoral vein; 1 hr later blood was collected from the abdominal aorta using 3.8% trisodium citrate as anticoagulant. An aliquot of the blood sample was diluted 200-fold with buffer (10 mM HEPES-Na, 136 mM NaCl, 2.7 mM KCl, 2.0 mM MgCl_2_, 1.0 mM NaH_2_PO_4_, 5.0 mM dextrose, 5 mg/mL BSA, 2.5 mM CaCl_2_, pH 7.4) and stained with annexin V-FITC for 15 min in the dark. PS exposure was measured as described above.

Venous thrombosis was induced by stasis combined with hypercoagulability. Rats (180–250 g body weight) were anesthetized with intraperitoneal urethane (1.25 g/kg), the abdomen was surgically opened, and the vena cava was exposed. Two loose cotton threads were prepared around the vena cava 16 mm apart, and all side branches were ligated tightly with cotton threads. One hour after intravenous injection of saline or HgCl_2_ (bolus, 0.25, 0.5, or 1.0 mg/kg/0.3 mL) into a left femoral vein, 1,000-fold diluted thromboplastin was infused to induce thrombus formation. Stasis was initiated by tightening the two threads, first the proximal one, and 30 sec later the distal one. The abdominal cavity was provisionally closed, and blood stasis was maintained for 15 min. After the abdomen was reopened, the ligated venous segment was excised and opened longitudinally to remove the thrombus. The isolated thrombus was blotted of excess blood and immediately weighed.

### Statistical analysis

We calculated mean ± SE for all treatment groups. The data were subjected to one-way analysis of variance followed by Duncan’s multiple range test to determine which means were significantly different from control. In all cases, *p* < 0.05 indicated significance.

## Results

**Figure 1:**
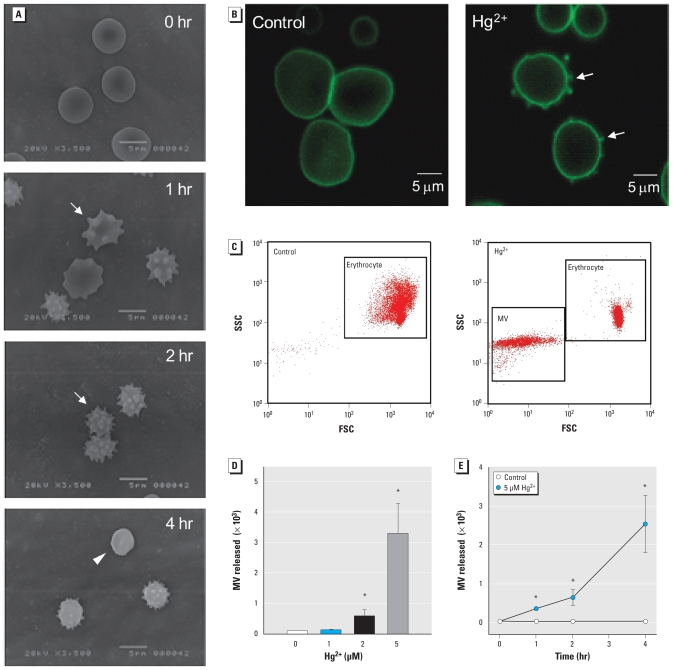
Effects of mercury on erythrocytes. (*A*) Photomicrographs showing shape changes in human erythrocytes incubated with 5 μM Hg^2+^ up to 4 hr at 37°C, fixed, and examined under SEM. Photomicrographs represent three independent experiments from different blood donors; bars = 5 μm. Arrows indicate echinocytes, and the arrowhead indicates spherocyte. (*B*) Photomicrographs of erythrocytes attached to poly-l-lysine–coated coverslip chambers, treated with TBS (control; left) or Hg^2+^ (5 μM; right) for 1 hr, and observed under confocal microscopy using erythrocyte-specific anti–glycophorin-A–FITC. Arrows indicate MV generation on erythrocyte membranes. (*C*) Forward scatter characteristics (FSC) versus side scatter characteristics (SSC) of erythrocytes incubated with distilled water (control) or Hg^2+^ for 4 hr at 37°C; erythrocytes and MV were identified in a representative dot plot (Hg^2+^ 5 μM). Time-dependent (*D*) and concentration-dependent (*E*) increases in MV generation in erythrocytes after Hg^2+^ incubation. Values are mean ± SE of three to five independent experiments. *p < 0.05 compared with control.

To investigate the effects of mercury on erythrocytes, we examined the shape change in erythrocytes after exposure to 5 μM HgCl_2_ using SEM. As shown in Figure 1A, normal discocytic shapes changed into echinocytic erythrocytes and further into spherocytes, depending on the length of exposure to Hg^2+^. It is well known that erythrocytes change into spherocytes by a substantial loss of membrane surface through vesiculation and MV generation. To visually identify MV generation, we treated erythrocytes attached to a poly-l-lysine–coated coverslip chamber with Hg^2+^ and observed them under confocal microscopy using erythrocyte-specific anti–glycophorin-A–FITC. Hg^2+^ treatment induced typical MV generation on erythrocyte membranes (Figure 1B, right). In flow cytometry analysis, MV generation increased in a time- and concentration-dependent manner after Hg^2+^ incubation (Figure 1C,D).

**Figure 2:**
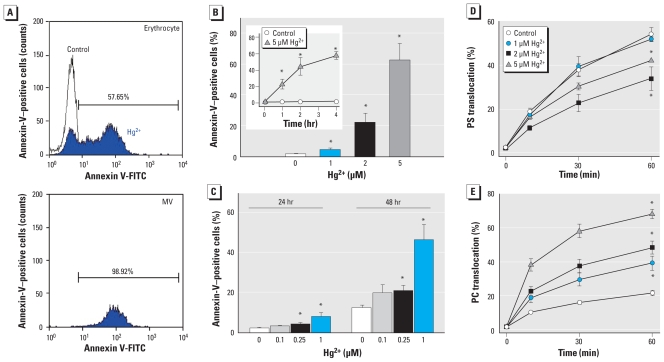
Effects of Hg^2+^ on PS exposure in MV and remnant erythrocytes and on aminophospholipid translocation. Human erythrocytes were incubated with distilled water (control) or Hg^2+^ at 37°C, and flow cytometric analysis was performed. (*A*) Representative histogram of remnant cells (top) and MVs (bottom) expressing PS, with annexin-V as a marker of PS positivity. (*B*, *C*) Time- and concentration-dependent effects of Hg^2+^ on PS exposure on erythrocytes incubated with distilled water (control) or 1, 2, or 5 μM Hg^2+^ for 1 hr at 37°C (*B*) or at varied Hg^2+^ doses for 24 or 48 hr (*C*). C_6_-NBD-PS (PS) translocation by flippase (*D*) and C_6_-NBD-PC (PC) translocation by scramblase (*E*). Values are mean ± SE of three independent experiments. *p < 0.05 compared with control.

MVs bearing PS can display procoagulant activity. In the flow cytometry analysis with annexin V-FITC to specifically detect PS, MVs generated by Hg^2+^ treatment expressed PS in their outer membranes (Figure 2A). Moreover, remnant erythrocytes also expressed PS in the outer membrane (62.2 ± 11.2% at 5 μM Hg^2+^; Figure 2B), consistent with the report by Eisele et al. (2006). PS expression also increased in a time-dependent manner with Hg^2+^ (Figure 2B, inset), and of particular note, prolonged exposure induced significant PS expression even at lower concentrations of Hg^2+^ (down to 0.25 μM; Figure 2C). To investigate the mechanism underlying Hg^2+^-induced PS exposure and MV generation, we evaluated the activities of representative aminophospholipid translocases governing lipid asymmetry. We measured flippase, an enzyme that recovers PS into the inner leaflet of the cell membrane, and scramblase, an enzyme that disrupts lipid asymmetry in the cell membrane, based on the extent of C_6_-NBD-PS and C_6_-NBD-PC translocation, respectively, after incubation of Hg^2+^. C_6_-NBD-PS and C_6_-NBD-PC are the fluorescent analogs of PS and phosphatidylcholine; we measured the internalization of C_6_-NBD-PS and C_6_-NBD-PC by flippase and scramblase, respectively, by comparing the fluorescence intensity associated with the cells before and after BSA back-extraction, as described in “Materials and Methods.” As shown in Figure 2D and E, flippase was inhibited by Hg^2+^, whereas scramblase was activated in a concentration-dependent manner, which explains Hg^2+^-induced PS exposure.

**Figure 3:**
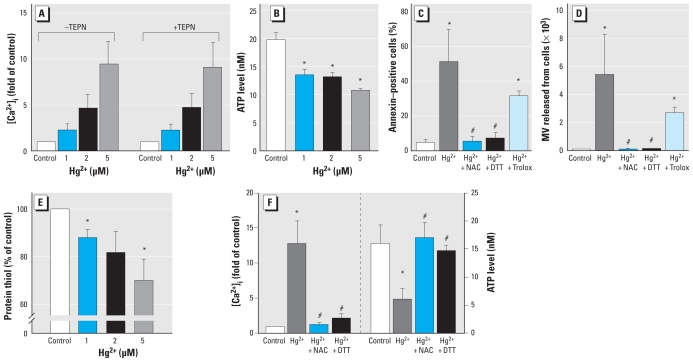
Effects of Hg^2+^ on intracellular Ca^2+^ ATP, and protein thiol in human erythrocytes. (*A*) Intracellular Ca^2+^ levels {[Ca^2+^]_i_}, in erythrocytes loaded with Fluo-4, incubated with distilled water (control) or 1, 2, or 5 μM Hg^2+^ 4 hr at 37°C, and evaluated by flow cytometry; 100 μM TPEN was used to exclude interference from intracellular Hg^2+^. (*B*) ATP level in erythrocytes incubated with distilled water (control) or 1, 2, or 5 μM Hg^2+^ for 4 hr at 37°C, and measured by a bioluminescent assay. PS exposure (*C*) and MV generation (*D*) measured by flow cytometry analysis in human erythrocytes pretreated with 0.5 mM N-acetylcysteine (NAC), 0.5 mM DTT (thiol supplements), or 100 μM Trolox (antioxidant) 5 min before treatment with 5 μM Hg^2+^. (*E*) Protein thiol measured using the DTNB method. (*F*) Inhibition of Ca^2+^ increase (left) and ATP depletion (right) by thiol supplements. Values are mean ± SE of three independent experiments. *p < 0.05 compared with control. ^#^*p* < 0.05 compared with Hg^2+^ treatment alone.

Increased intracellular calcium can mediate concomitant inhibition of flippase and activation of scramblase, leading to the disruption of membrane lipid asymmetry (Daleke 2003). To investigate whether Hg^2+^ treatment can induce calcium increase, we incubated Fluo-4–loaded erythrocytes with Hg^2+^ and measured intracellular calcium increase by flow cytometry analysis. As shown in Figure 3A, Hg^2+^ treatment increased intracellular calcium prominently, up to 10 times the basal level. For this calcium signal, an artifactual increase due to possible interference with Fluo-4 binding by Hg^2+^ could be excluded by adding the Hg^2+^ quencher TPEN, a high-affinity membrane- permeable intracellular heavy metal chelator (Arslan et al. 1985). ATP depletion, which can prompt flippase inhibition, was also induced by Hg^2+^ (Figure 3B). In general, Hg^2+^-induced cytotoxicity is mediated by thiol depletion (Zalups 2000) or oxidative stress (Wiggers et al. 2008). To examine the role of thiol depletion and oxidative stress in Hg^2+^-induced PS exposure and MV generation, we preincubated erythrocytes with the thiol supplements N-acetylcysteine and dithiothreitol (DTT) or the antioxidant Trolox. As shown in Figure 3C, Trolox was marginally effective, whereas thiol supplements effectively blocked Hg^2+^-induced PS exposure and MV generation, indicating a major role of thiol depletion. Indeed, Hg^2+^ treatment induced significant thiol depletion (Figure 3E). In line with these findings, thiol supplementation prevented Hg^2+^-induced ATP depletion and Ca^2+^ increase (Figure 3F), indicating that Hg^2+^ induces PS exposure and MV generation in erythrocytes through protein-thiol depletion–mediated ATP depletion and Ca^2+^ increases.

**Figure 4:**
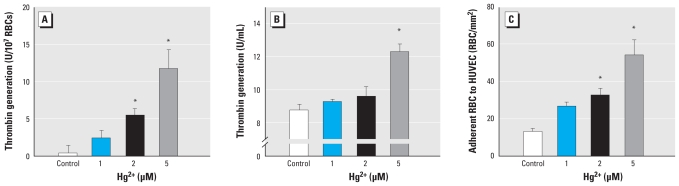
Enhancement of thrombin generation and erythrocyte adherence to endothelial cells by Hg^2+^ treatment. (*A*) Erythrocytes (red blood cells, RBCs) were incubated with distilled water (control) or 1, 2, or 5 μM Hg^2+^ for 4 hr at 37°C, and aliquots were subjected to the prothrombinase assay. (*B*) Hg^2+^-treated erythrocytes were added to human plasma, and thrombin generation was initiated by Recombiplastin. (*C*) Hg^2+^-treated erythrocytes were incubated with HUVECs for 45 min; after washing, erythrocytes adhered to HUVECs were counted under a microscope. Values are mean ± SE of three to five independent experiments. *p < 0.05 compared with control.

**Figure 5:**
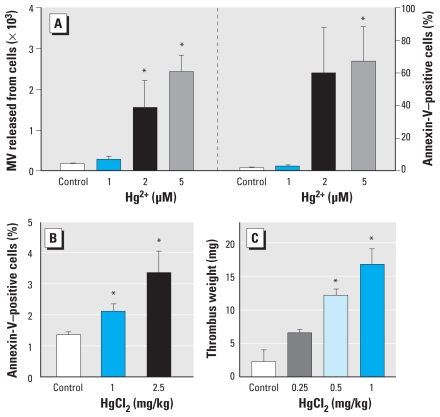
Effects of Hg^2+^ on PS exposure and thrombus formation in an *in vivo* rat model. (*A*) MV generation and PS exposure in rat erythrocytes (red blood cells, RBCs) treated with distilled water (control) or 1, 2, or 5 μM Hg^2+^ for 4 hr at 37°C and measured by flow cytometry. Extent of PS exposure on erythrocytes measured in whole blood using flow cytometry (*B*), and thrombus formation induced by the infusion of thromboplastin (*C*) in rats administered intravenous saline (control) or HgCl_2_ (0.25–2.5 mg/kg). Values are the mean ± SE of four independent experiments. *p < 0.05 compared with control.

We confirmed prothrombotic effects of Hg^2+^-induced PS exposure and MV generation by evidence of increased thrombin generation based on the prothrombinase assay and thrombin generation in plasma (Figure 4A,B). Moreover, we demonstrated increased adherence of Hg^2+^-exposed erythrocytes to endothelial cells by increased erythrocyte adhesion on HUVEC (Figure 4C). To evaluate the *in vivo* relevance of Hg^2+^-induced procoagulant activity in erythrocytes, we used rat *ex vivo* PS exposure measurement and *in vivo* venous thrombosis models. Before the *in vivo* experiments, we confirmed PS exposure and MV generation by Hg^2+^ in rat erythrocytes (Figure 5A), which showed a pattern similar to that of human erythrocytes. Confirming these *in vitro* results, treatment of HgCl_2_ [intravenous bolus 0.25–2.5 mg/kg (0.92–9.2 μmol/kg)] induced PS exposure on erythrocytes *ex vivo* (Figure 5B; 2.1 ± 0.2% for 1 mg/kg and 3.4 ± 0.7% for 2.5 mg/kg HgCl_2_ vs. 1.4 ± 0.1% for vehicle; *p* < 0.05) and promoted clot formation in a stasis- and hypercoagulability-induced venous thrombosis model after thromboplastin infusion (Figure 5C; 12.2 ± 1.0 mg for 0.5 mg/kg HgCl_2_ and 16.9 ± 2.3 mg for 1 mg/kg HgCl_2_ vs. 2.4 ± 1.7 mg for vehicle; *p* < 0.01), suggesting that Hg^2+^-induced procoagulant activity in erythrocytes could contribute to increased thrombosis *in vivo*.

## Discussion

**Figure 6:**
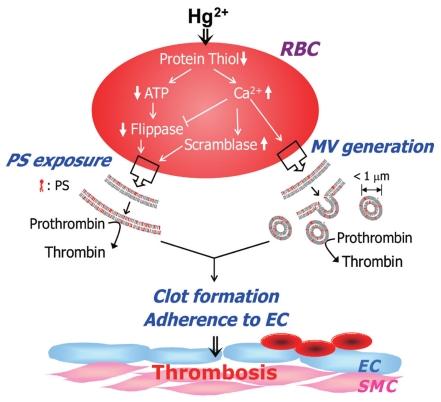
Suggested mechanism of Hg^2+^-induced procoagulant activation of erythrocytes. Hg^2+^ induces procoagulant activation of erythrocytes and enhanced thrombus formation through thrombogenic PS exposure and MV generation mediated by protein thiol depletion, ATP depletion, and Ca^2+^ increase via scramblase activation and flippase inhibition. Abbreviations: EC, endothelial cell; RBC, red blood cells (erythrocytes); SMC, smooth muscle cell.

In this study, we demonstrated that Hg^2+^ can induce shape changes, MV generation, and PS exposure on erythrocytes, important mediators of procoagulant activation of erythrocytes. Hg^2+^-mediated thiol depletion was responsible for calcium increase–mediated and ATP depletion–mediated flippase inhibition and scramblase activation, leading to increased thrombin generation, enhanced adhesion of erythrocytes to endothelial cells, and ultimately, accelerated thrombus formation (Figure 6). Of particular note, *ex vivo* PS exposure measurement and the rat *in vivo* venous thrombosis model confirmed the procoagulant effect of Hg^2+^ by demonstrating increased PS exposure and clot formation, suggesting that procoagulant activation of erythrocytes might contribute to CVDs associated with mercury exposure.

Erythrocytes can contribute to hemostasis and thrombosis through procoagulant activation via PS exposure and MV generation (Zwaal et al. 2005; Zwaal and Schroit 1997). Endogenous thrombogenic substances such as arachidonic acid, lysophosphatidic acid, and thromboxane are known to induce PS exposure on erythrocyte surfaces (Chung et al. 2007; Valles et al. 2002). In addition, we previously demonstrated that lead, another toxic heavy metal, can induce a substantial level of PS exposure in erythrocytes and that this procoagulant activity manifested as enhanced thrombus formation (Shin et al. 2007). Interestingly, it is well known that erythrocytes can be a preferential store for toxic heavy metals. Methylmercury accumulates in erythrocytes at a concentration > 20 times that of plasma (Clarkson 2002), and 90% of body lead is associated with erythrocytes. These findings suggest that erythrocytes can be a common target for CV toxicity of heavy metals; thus, the role of erythrocytes in CV toxicity of heavy metals should be more closely investigated.

Millimolar concentrations of Hg^2+^ have been reported to induce shape changes and hemolysis in erythrocytes (Suwalsky et al. 2000; Zolla et al. 1997). The concentration employed in those studies, however, was unrealistically high, considering reported human exposure levels even in highly contaminated areas. In the present study, however, we discovered that longer exposure to concentrations of Hg^2+^ as low as 0.25 μM can induce shape changes and procoagulant activation in erythrocytes as determined by increased PS exposure. The highest blood mercury concentrations reported in humans were in workers in the gold mines of the Amazon area (Akagi et al. 1995), whose blood mercury levels were as high as 150 μg/L (~ 0.75 μM). Wierzbicki et al. (2002) reported that workers occupationally exposed to mercuric vapors exhibited a statistically significant increase in blood coagulation along with increased thrombin generation. Their average plasma mercury levels were approximately 0.03–0.04 μM but measured as high as 83.3 μg/L (0.4 μM). Virtanen et al. (2005) observed increased CV events in a mercury-exposed population with an average hair mercury level of 1.9 μg/g (0–15.7 μg/g), which is estimated to indicate a blood mercury level of 9.5 μg/L (0.05 μM) based on a 200:1 hair-to-blood mercury ratio (Budtz-Jorgensen et al. 2004). These levels are within an order of magnitude of the mercury concentrations used in our study, suggesting that mercury-induced procoagulant activation of erythrocytes might indeed contribute to the increased CVDs in human populations. However, because we conducted our study with only Hg^2+^, further *in vivo* studies with methylmercury or elemental mercury are needed to fully clarify the link between mercury-mediated procoagulant activation of erythrocytes and mercury-associated CVDs.

Adding to the previous report on Hg^2+^-induced echinocytic changes in human erythrocytes (Suwalsky et al. 2000), we found that echinocytes can be further progressed into spherocytes (Figure 1A) by prolonged exposure to Hg^2+^. Moreover, along with these remarkable shape changes, substantial numbers of MVs can be liberated from erythrocytes. Apart from clot-promoting activity in plasma, MV generation can result in decreased deformability of erythrocytes (Chabanel et al. 1989; Chasis and Mohandas 1986; Mentzer et al. 1987) and, more important, PS-exposing MVs provide sites for adhesion of platelets and neutrophils by localizing at the subendothelium, indicating that Hg^2+^-induced MV generation can contribute to the prothrombotic effects of mercury exposure.

In our study, we determined thiol depletion to be a key mediator for calcium increase, ATP depletion, PS exposure, and MV generation in erythrocytes by Hg^2+^. Thiol depletion by Hg^2+^ can be well explained by the strong thiol-binding affinity of nucleophilic Hg^2+^ (Casarett et al. 2007). Intracellular molecules with free sulfhydryl groups, such as glutathione, cysteine, and metallothionein, can be easy targets for Hg^2+^ binding, and free thiol groups of intracellular proteins, which are vital to the maintenance of erythrocyte integrity (e.g., of the cytoskeleton) and ionic balance (e.g., Na^2+^/K^+^-ATPase), can be readily modified by thiol-depleting agents, leading to a increased fragility of the erythrocyte membrane and disruption of ionic homeostasis. Moreover, depletion of free thiols in cells can cause depletion of ATP, inhibition of flippase (Daleke and Lyles 2000), and increased calcium, consistent with the result of the present study.

## Conclusion

We demonstrated that low-dose mercury can induce thrombogenic PS exposure and MV generation through thiol-depletion–mediated ATP depletion and calcium increase in erythrocytes. As shown by increased PS exposure and clot formation *in vivo*, mercury-induced procoagulant activation of erythrocytes might contribute to enhanced thrombosis in the population exposed to mercury, providing an important clue for the elucidation for the CVDs associated with mercury.
